# Author Correction: Electrostatic potentials of atomic nanostructures at metal surfaces quantified by scanning quantum dot microscopy

**DOI:** 10.1038/s41467-026-75450-6

**Published:** 2026-07-09

**Authors:** Rustem Bolat, Jose M. Guevara, Philipp Leinen, Marvin Knol, Hadi H. Arefi, Michael Maiworm, Rolf Findeisen, Ruslan Temirov, Oliver T. Hofmann, Reinhard J. Maurer, F. Stefan Tautz, Christian Wagner

**Affiliations:** 1 Peter Grünberg Institut (PGI-3), Forschungszentrum Jülich, 52425 Jülich, Germany; 2 Jülich Aachen Research Alliance (JARA), Fundamentals of Future Information Technology, 52425 Jülich, Germany; 3 Experimentalphysik IV A, RWTH Aachen University, Otto-Blumenthal-Straße, 52074 Aachen, Germany; 4 Control and Cyber-Physical Systems Laboratory, Technische Universität Darmstadt, 64277 Darmstadt, Germany; 5 II. Physikalisches Institut, Universität zu Köln, 50937 Köln, Germany; 6 Institute of Solid State Physics, NAWI Graz, Graz University of Technology, Petersgasse 16, 8010 Graz, Austria; 7 Department of Chemistry, University of Warwick, Gibbet Hill Road, Coventry, UK; 8 Department of Physics, University of Warwick, Gibbet Hill Road, Coventry, UK

**Keywords:** Chemical physics, Electronic properties and materials, Surfaces, interfaces and thin films

Correction to: *Nature Communications* 10.1038/s41467-024-46423-4, published online 13 March 2024

In the version of this article initially published, there were errors in Fig. 3a, b, where the colors in the figure and legend denoting Ag chains (now shown in teal) and Au chains (now shown in dark yellow) were interchanged. The figure and legend are now amended in the HTML and PDF versions of the article.

