# Supplementary material for: Electrostatic potentials of atomic nanostructures at metal surfaces quantified by scanning quantum dot microscopy
Source: Nat Commun. 2024 Mar 13;15:2259. doi: 10.1038/s41467-024-46423-4 (PMC10937982; doi:10.1038/s41467-024-46423-4)
Supplement: Supplementary file 1 — Supplementary Information [file 41467_2024_46423_MOESM1_ESM.pdf]

Supplementary information for
Electrostatic potentials of atomic nanostructures at metal surfaces quantified by scanning quantum dot microscopy

R. Bolat,[1,2,3] J. M. Guevara,[1] P. Leinen,[1] M. Knol,[1,2,3] H. H. Arefi,[1,2] M. Maiworm,[4] R. Findeisen,[4] R. Temirov,[1,2,5] O. T. Hofmann,[6] R. J. Maurer,[7,8] F. S. Tautz,[1,2,3] and C. Wagner[1,2]

[1] *Peter Grünberg Institut (PGI-3), Forschungszentrum Jülich, 52428 Jülich, Germany*
[2] *Jülich Aachen Research Alliance (JARA), Fundamentals of Future Information Technology, 52425 Jülich, Germany*
[3] *Experimentalphysik IV A, RWTH Aachen University, Otto-Blumenthal-Straße, 52074 Aachen, Germany*
[4] *Control and Cyber-Physical Systems Laboratory, Technische Universität Darmstadt, 64277 Darmstadt, Germany*
[5] *II. Physikalisches Institut, Universität zu Köln, 50937 Köln, Germany*
[6] *Institute of Solid State Physics, NAWI Graz, Graz University of Technology, Petersgasse 16, 8010 Graz, Austria*
[7] *Department of Chemistry, University of Warwick, Gibbet Hill Road, Coventry, UK*
[8] *Department of Physics, University of Warwick, Gibbet Hill Road, Coventry, UK*

To ensure that our computed data are correct, we have carefully converged the numerical settings to make sure the results are converged within 0.05 D.

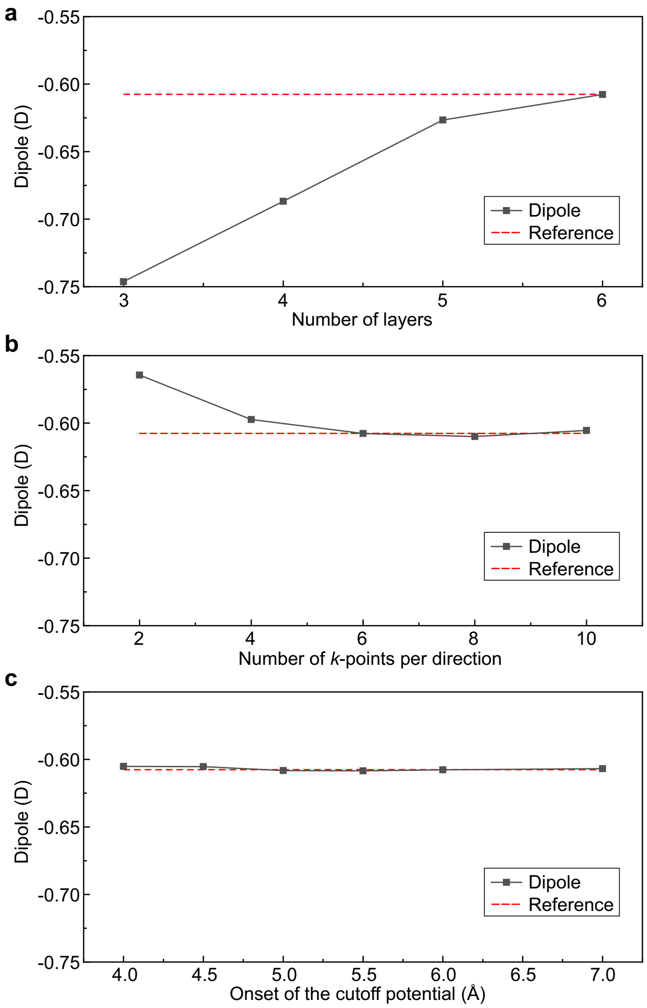

Supplementary Figure 1. **Convergence of DFT calculation.** Convergence of the DFT-computed dipole moment with respect to **(a)** the number of layers used for the metal slab; **(b)** the number of k-points used to model the unit cell, and **(c)** the confining cutoff potential for the numerically tabulated basis functions, as used in FHI-aims. Shown are the results for a single Ag adatom on the Ag(111) surface (grey). The dashed red line shows the result used in the main text.
